# Increased platelet distribution width predicts poor prognosis in melanoma patients

**DOI:** 10.1038/s41598-017-03212-y

**Published:** 2017-06-07

**Authors:** Na Li, Zhiyong Diao, Xiaoyi Huang, Ye Niu, Tiemin Liu, Zhi-ping Liu, Rui-tao Wang, Kai-jiang Yu

**Affiliations:** 1 Department of Internal Medicine, Harbin Medical University Cancer Hospital, Harbin Medical University, Harbin, Heilongjiang 150081 China; 2 Department of Plastic Surgery, the First Affiliated Hospital, Harbin Medical University, Harbin, Heilongjiang 150001 China; 3 Biotherapy Center, Harbin Medical University Cancer Hospital, Harbin Medical University, Harbin, Heilongjiang 150081 China; 4 Department of Geriatrics, the Second Affiliated Hospital, Harbin Medical University, Harbin, Heilongjiang 150086 China; 5 Division of Hypothalamic Research, Department of Internal Medicine, UT Southwestern Medical Center, Dallas, TX 75390 USA; 6 Departments of Internal Medicine and Molecular Biology, University of Texas Southwestern Medical Center, Dallas, TX USA; 7 Department of Intensive Care Unit, Harbin Medical University Cancer Hospital, Harbin Medical University, Harbin, Heilongjiang 150081 China

## Abstract

Activated platelets promote cancer progression and metastasis. Nevertheless, the prognostic value of platelet indices in melanoma had been rarely reported. The aim of this study was to investigate the predictive significance of platelet indices in melanoma. A total of 220 consecutive patients with melanoma were retrospectively enrolled between January 2009 and December 2009. The relationship between PDW and clinicopathological characteristics were analyzed. Kaplan-Meier method and Cox regression were used to evaluate the prognostic impact of PDW. Of the 220 patients, high platelet distribution width (PDW) levels were observed in 63 (28.6%) patients. Increased PDW was associated with tumor subtype (P < 0.001). Survival curves found that patients with increased PDW had significantly shorter survival time than those with normal PDW (P < 0.001). Cox regression analysis revealed that elevated PDW was an independent prognostic factor for overall survival (hazard ratio, 2.480; 95% confidence interval [CI], 1.386–4.436, P = 0.002). In conclusion, PDW is easily available in routine blood test. Our findings indicated that PDW is an independent predictor and that it may also be a potential parameter for targeted therapy in melanoma.

## Introduction

Malignant melanoma is an aggressive form of cancer with an increasing incidence and mortality worldwide. Despite multiple and aggressive therapeutic interventions, some patients still recur after treatment. Therefore, it is of great importance to look for appropriate and effective prognostic markers in melanoma.

Platelets play an essential role in cancer development, progression and metastasis though their direct interaction with tumor cell^1^. Platelet actions trigger autocrine and paracrine activation processes that cause phenotypic changes in stromal cells which contribute to the development of cancer^2^. Increased platelets were associated with poor prognosis in patients with a wide spectrum of malignancies, such as pancreatic cancer, gastric cancer, colorectal cancer, endometrial cancer, and ovarian cancer^3–7^. However, platelet count is determined by the balance between the rate of production and consumption of platelets. A normal platelet count could conceal the presence of highly hypercoagulative and pro-inflammatory cancer phenotypes in the presence of efficient compensatory mechanisms^8^.

Mean platelet volume (MPV), the most commonly used measure of platelet size, is an index of platelet activation and is available in clinical practice^9^. Platelet distribution width (PDW), another platelet index, indicates variation in platelet size^10^. Altered MPV levels were reported in gastric cancer, ovarian cancer, lung cancer, colon cancer, and breast cancer. However, the clinical implications of PDW have not been well defined. In the current study, therefore, we aimed to evaluate the prognostic roles of MPV and PDW in patients with melanoma.

## Results

Between Jan, 2009 and Dec, 2009, a total of 220 patients were enrolled in this study. Among the 220 patients, 116 (52.7%) were women and 104 (47.3%) were men, and the median age was 56.3 ± 12.4 years (range 21–86). In terms of the staging system, 36 cases were categorized as stage I and stage II, 129 as stage III and stage IV.

**Figure 1:**
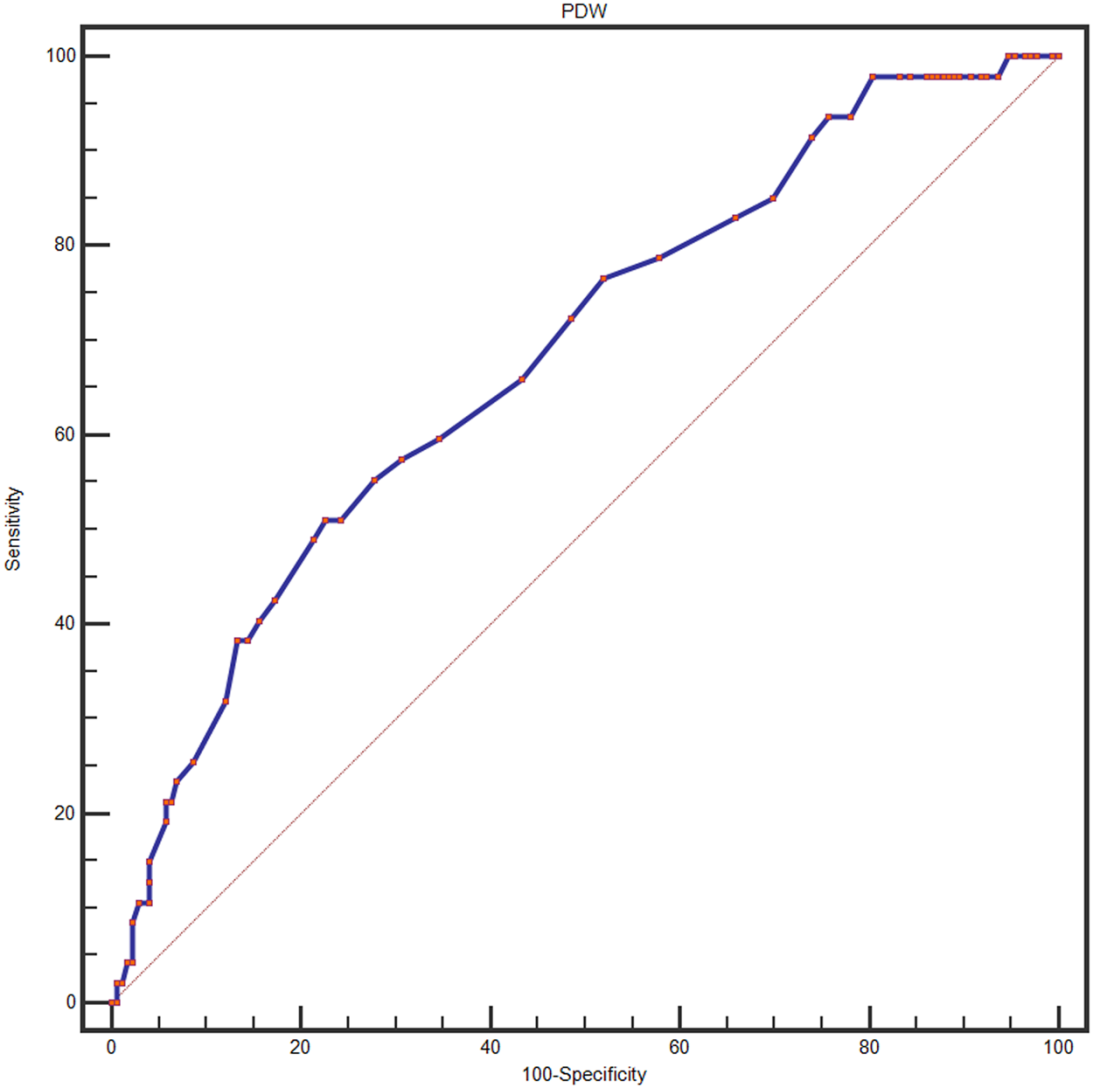
Optimized cut-off was determined for PDW using standard ROC curve analysis.

A ROC curve for OS prediction was plotted to verify the optimal cut-off value for PDW, which was 17.2 (Fig. 1). It demonstrated that PDW predicts cancer prognosis with a sensitivity of 51.1% and a specificity of 68.3% (AUC = 0.683, 95% CI: 0.618–0.744, p < 0.0001). Then, patients were divided into 2 groups: patients with PDW ≤ 17.2% and patients with PDW > 17.2%. There were 157 (71.4%) patients with PDW ≤ 17.2% and 63 (28.6%) patients with PDW > 17.2%.

The relationships between PDW and clinical characteristics were shown in Tables 1 and 2. Our study revealed that PDW was associated with tumor subtype (P < 0.001). However, no significant differences were observed between the groups with regard to age, gender, tumor location, ulceration, tumor size, lymph node metastasis, distant metastasis, and clinical stage.

**Table 1:** Baseline characteristics of melanoma patients according to PDW levels.

Variables	Total n (%)	PDW ≤ 17.2 n (%)	PDW > 17.2 n (%)	P value
Age (years)				0.849
<60	127 (57.7)	90 (57.3)	37 (58.7)	
≥60	93 (42.3)	67 (42.7)	26 (41.3)	
Gender				0.119
Male	104 (47.3)	69 (43.9)	35 (55.6)	
Female	116 (52.7)	88 (56.1)	28 (44.4)	
Tumor location				0.728
Sun-exposed (head and neck)	22 (10.0)	15 (9.6)	7 (11.1)	
Sun-protected (others)	198 (90.0)	142 (90.4)	56 (88.9)	
Ulceration				0.091
Negative	138 (62.7)	93 (59.2)	45 (71.4)	
Positive	82 (37.3)	64 (40.8)	18 (28.6)	
Tumor Size				0.187
≥2 mm	127 (57.7)	95 (60.5)	32 (50.8)	
<2 mm	93 (42.3)	62 (39.5)	31 (49.2)	
Tumor subtype				<0.001
ALM	89 (40.5)	66 (42.0)	23 (36.5)	
SSM	65 (29.5)	45 (28.7)	20 (31.7)	
LMM	26 (11.8)	17 (10.8)	9 (14.3)	
NM	29 (13.2)	24 (15.3)	5 (7.9)	
Others	11 (5.0)	5 (3.2)	6 (9.5)	
Lymph node metastasis				0.116
Negative	166 (75.0)	123 (78.3)	43 (68.3)	
Positive	54 (25.0)	34 (21.7)	20 (31.7)	
Distant metastasis				0.366
Absent	192 (87.3)	135 (86.0)	57 (90.5)	
Present	28 (12.7)	22 (14.0)	6 (9.5)	
Clinical stage				0.192
I/II	157 (71.4)	116 (73.9)	44 (65.1)	
III/IV	63 (28.6)	41 (26.1)	22 (34.9)	

SSM, superficial spreading melanoma; LMM, lentigo maligna melanoma; ALM, acrolentigous melanoma; NM, nodular melanoma; PDW, platelet distribution width.

**Table 2:** Baseline characteristics of melanoma patients according to PDW levels.

Variables	PDW ≤ 17.2	PDW > 17.2	P value
Age (years)	56.2 (12.6)	56.5 (12.2)	0.834
Smoker (n, %)	17 (10.8)	13 (20.6)	0.570
Drinking (n, %)	14 (8.9)	16 (25.4)	0.525
BMI (kg/m^2^)	24.8(3.4)	23.6 (2.9)	0.007
FPG (mmol/L)	5.10 (4.80–5.62)	5.00 (4.70–5.50)	0.444
Albumin (g/L)	43.3 (4.3)	44.6 (4.5)	0.047
WBC (×10^9^/L)	6.44 (2.05)	5.94 (1.87)	0.095
Neutrophils (×10^9^/L)	3.84 (1.74)	3.49 (1.65)	0.173
Lymphocytes (×10^9^/L)	2.07 (1.36)	1.85 (0.58)	0.239
Hemoglobin (g/dl)	138.8 (29.8)	139.3 (17.4)	0.903
Platelet count (×10^9^/L)	242.2 (67.7)	217.3 (60.5)	0.011
MPV (fL)	8.6 (1.2)	9.2 (1.5)	0.001
NLR	2.12(1.32)	2.15(2.30)	0.904
PLR	133.8 (58.9)	125.4 (46.2)	0.265

Data are expressed as means (SD) or median (IQR). FPG, fasting plasma glucose; WBC, white blood cell; BMI, body mass index; MPV, mean platelet volume; PDW, platelet distribution width; NLR, neutrophil-to-lymphocyte ratio; PLR, platelet-to-lymphocyte ratio.

**Figure 2:**
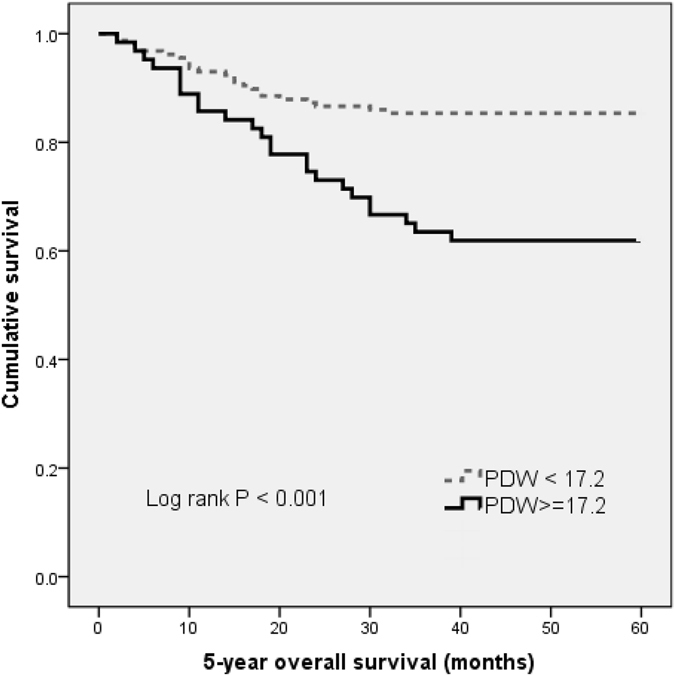
Kaplan–Meier analysis of overall survival in melanoma patients.

With a median follow up of 60 months, 47 (21.4%) patients had death events. Patients with PDW ≤ 17.2% had a significantly better 5-year OS than patients with PDW > 17.2% (85.4% vs. 61.9%, P < 0.001). The Kaplan-Meier OS curves of the normal versus elevated PDW showed a significant separation (Fig. 2).

In univariate analysis, lymph node metastasis, PDW (categorical variable), albumin, and clinical stage were significant predictors of OS (Table 3). Age (categorical variable) (p = 0.093), lymphocytes (p = 0.071), and tumor subtype (p = 0.075) showed weak associations. Other parameters were not found to be in correlation with OS. Next, all the factors with a P value less than 0.05 in univariate analysis were included in multivariate analysis (Table 4). In multivariate analyses, we demonstrated that PDW was an independent prognostic factor in patients with melanoma. Patients with PDW > 17.2% had a hazard ratio (HR) of 2.480 [95% confidence interval (CI): 1.386–4.436, P = 0.002] for OS.

**Table 3:** Univariate analysis of overall survival in melanoma patients.

	Hazard ratio	95% CI	*P*-value
Age (years) (≥60 versus <60)	0.591	0.320–1.092	0.093
Gender (male versus female)	0.769	0.433–1.364	0.368
Smoker (yes versus no)	1.060	0.257–4.368	0.936
Drinking (yes versus no)	0.423	0.152–1.180	1.000
BMI (kg/m^2^)	0.945	0.865–1.033	0.215
FPG (mmol/L)	1.360	0.897–2.063	0.148
Albumin (g/L)	1.075	1.009–1.145	0.026
WBC (×10^9^/L)	0.894	0.756–1.057	0.189
Neutrophils (×10^9^/L)	0.931	0.766–1.133	0.477
Lymphocytes (×10^9^/L)	0.612	0.359–1.042	0.071
NLR	1.105	0.941–1.298	0.224
PLR	1.004	0.978–1.013	0.613
Hemoglobin (g/dl)	0.996	0.983–1.009	0.562
Platelet count (×10^9^/L)	1.001	0.997–1.005	0.681
MPV (fL)	0.918	0.737–1.143	0.444
PDW (%) (>17.2 versus ≤17.2)	2.857	1.612–5.063	<0.001
Tumor location (Sun-exposed versus Sun-protected)	1.721	0.771–3.843	0.185
Ulceration (Yes versus No)	0.675	0.361–1.261	0.218
Tumor subtype (SSM + NM versus ALM + LMM + others)	0.592	0.332–1.055	0.075
Tumor Size (mm) (≥2.0 versus<2.0)	1.102	0.616–1.974	0.743
Lymph node metastasis (Yes versus No)	2.889	1.625–5.136	<0.001
Distant metastasis (Yes versus No)	1.219	0.546–2.721	0.629
Clinical stage (III/IV versus I/II)	3.386	1.908–6.009	<0.001

Abbreviations: see to Tables 1 and 2.

**Table 4:** Multivariate analysis of overall survival in melanoma patients.

	Hazard ratio	95% CI	*P*-value
Albumin (g/L)	1.051	0.985–1.122	0.131
PDW (%) (>17.2 versus ≤17.2)	2.480	1.386–4.436	**0.002**
Lymph node metastasis (Yes versus No)	0.660	0.223–1.953	0.453
Clinical stage (III/IV versus I/II)	4.311	1.479–12.568	**0.007**

CI, confidence interval. Abbreviations: see to Tables 1 and 2.

## Discussion

This study showed that PDW is associated with patient’s survival and is an independent risk factor for prognosis in melanoma.

Platelets facilitate cancer progression and metastasis by inducing tumor growth, epithelial-mesenchymal transition, and invasion^11^. An increasingly body of evidence have identified the involvement of activated platelets in melanoma. Platelet-derived growth factor (PDGF) secreted by melanoma cell could stimulate the development of tumor stroma and new blood vessels^12^. Moreover, Boukerche H *et al*. showed platelet-melanoma cell interaction is mediated by the glycoprotein IIb-IIIa complex^13^. Kolber DL *et al*. confirmed that recombinant platelet factor 4, a known angiogenesis inhibitor, could effectively suppress tumor-induced neovascularization in mice^14^. In accordance with the studies above, the current study indirectly confirmed the findings using a simple platelet index. These data are also consistent with the current knowledge that anti-platelet is considered to be a part of cancer adjuvant therapy^1^. In addition, our study can form the basis for further mechanistic studies and ultimately aid in patient-tailored selection of therapeutic strategies.

The mechanisms to explain the association between PDW and survival are poorly understood. Bone marrow cells (including megakaryocytes) dys-function may contribute to altered PDW. PDW is a measure of platelet heterogeneity caused by heterogeneous demarcation of megakarocytes^15^. Recent reports demonstrated several cytokines, such as interleukin-6 (IL-6), granulocytes colony stimulating factor (G-CSF) and macrophage colony stimulating factor (M-CSF), regulate megakaryocytic maturation, platelet production and platelet size^16^. IL-6 promotes tumor angiogenesis, metastasis and metabolism^17^. Furthermore, the cytokines G-CSF and M-CSF that be secreted by tumor cells could stimulate megakaryopoiesis and subsequent thrombopoiesis in cancer^18^. However, the clinical value of PDW has not been studied in melanoma. Another possible mechanism is that platelets promote the hypercoagulable state in cancer^19^. Activated platelets create a procoagulant micro-environment that enables the tumor cells to cover themselves with platelets and evade the host immune system^20^.

The present study has several limitations. First, this was a single-center retrospective study and additional larger validation studies with multiethnic groups are needed to confirm our results. Second, the mechanisms underlying the involvement of PDW in melanoma remains unclear, to which further investigation should be addressed. Third, the patients were composed of Chinese. The application to other ethnic groups still needs further investigation.

In conclusion, PDW is easily available with routine blood counts. Increased PDW may serve as a marker of adverse prognosis in melanoma. Further studies are warranted to clarify the exact role of PDW in melanoma.

## Patients and Methods

### Study population

This study consisted of 220 consecutive melanoma cases (mean age 56.3 ± 17.4 years, range 18–86 years). Cases were admitted to the Third Affiliated Hospital, Harbin Medical University between January 2009 and December 2009. All patients undergone complete surgical resection. The pathologic diagnoses of melanoma were evaluated by pathologists from biopsy reports. None of the patients received preoperative chemotherapy or radiation therapy. Patients were excluded if they had hematological disorders, coronary artery disease, hypertension, diabetes mellitus, and medical treatment with anticoagulant, statins, and acetylic salicylic acid.

Standard demographic and clinicopathological data were collected from the patients’ records in hospital. For all the study participants, venous peripheral blood samples were collected at admission. Survival data were obtained through follow-up. Overall survival (OS) was defined as the interval from the date of diagnosis to death or last follow-up. The median follow-up time was 60 months. The platelet-to-lymphocyte ratio (PLR) was calculated as the absolute platelet count measured in ×10^9^/L divided by the absolute lymphocyte count measured in ×10^9^/L. The neutrophil-to-lymphocyte ratio (NLR) was calculated as the absolute neutrophil count measured in ×10^9^/L divided by the absolute lymphocyte count measured in ×10^9^/L.

The Institutional Ethics Review Board of the 3^rd^ Affiliated Hospital of Harbin Medical University approved this study prior to commencement of data collection and waived the informed consent requirement because it was a retrospective study.

### Statistical analysis

All statistical analyses were performed using SPSS Statistics version 22.0 (SPSS Inc., Chicago, IL, USA). The descriptive statistics are presented as means ± SD or medians (interquartile range) for continuous variables and percentages of the number for categorical variables. Inter-group differences in categorical variables were assessed for significance using the Chi-square test; differences in continuous variables were assessed using the Mann-Whitney U test or t-test. The optimal cutoff value of PDW was determined by receiver operating characteristic (ROC) curve. We used univariate analysis to narrow down the list of possible prognostic factors. Variables with P value < 0.05 in univariate analysis were brought into multivariate Cox proportional hazard model to determine their independency. Kaplan-Meier curves and log-rank test were used to compare survival differences among groups. All reported p-values are two-sided and statistical significance was assumed as p < 0.05.
